# Seasonal variations in biomass, height, photosynthetic efficiency, and carbon and nitrogen contents of *Suaeda japonica* in Incheon salt marshes (Korea)

**DOI:** 10.3389/fpls.2025.1513624

**Published:** 2025-03-11

**Authors:** Jeong Hwa Hwang, Ji-Sook Park, Young-Seok Han, Charles Yarish, Jang K. Kim

**Affiliations:** ^1^ Department of Marine Science, Incheon National University, Incheon, Republic of Korea; ^2^ Research Institute of Basic Sciences, Incheon National University, Incheon, Republic of Korea; ^3^ NEB Geumcheon, Seoul, Republic of Korea; ^4^ Department of Ecology and Evolutionary Biology, University of Connecticut, Storrs, CT, United States

**Keywords:** salt marsh plant, *Suaeda japonica*, carbon sequestration, photosynthesis, Incheon

## Abstract

Salt marshes are known as major blue carbon ecosystems for their higher carbon sequestration capacity and productivity compared to terrestrial ecosystems. However, in Korea, salt marshes have been continuously decreasing since the 1980s. This study aims to identify seasonal changes in salt marsh plants and evaluate the carbon and nitrogen sequestration in these ecosystems. This study observed seasonal changes in the biomass, cover, height, photosynthetic efficiency, carbon and nitrogen content of *Suaeda japonica* in two salt marsh ecosystems in Incheon, Korea, Yeongjong (YJ) and Sorae (SR). In spring, plant density at SR. was significantly higher than at YJ. This higher density at SR inhibited growth and survival during summer and autumn. In addition, photosynthetic efficiency at SR decreased significantly in summer compared to spring. The different habitat densities between the two sites resulted in morphological variations. The plants at YJ, with lower density, grew wider with more branches and showed higher dry weight in comparison to those at SR. Carbon sequestration by *S. japonica* per unit area was 113.70 g/m^2^ at YJ, which was twice as high as at SR. The plant density and biomass of *S. japonica* were affected by differences in seawater inflow at each site. The tissue carbon content was highest in the roots, suggesting that carbon absorbed by the leaves is effectively stored in sediment. This study provides insights into the regional and seasonal changes of *S. japonica*, highlighting its significance as a blue carbon resource. The results can contribute to the evaluation and restoration of salt marshes to enhance their carbon sequestration potential.

## 1 Introduction

Among these wetlands, the place locates near the coastline is called salt marsh. Salt marshes cover less than 1% of the Earth’s coastal regions, from polar to tropical areas, and exhibit high primary productivity (Chmura et al., 2003; Ewers Lewis et al., 2018; Hopkinson et al., 2012). The marshes are known to span an area of 22,000 km^2^ to a maximum of 400,000 km^2^ globally (Chmura et al., 2003; Hopkinson et al., 2012). However, these wetland ecosystems have gradually transitioned to terrestrial habitats by human activities such as agriculture and urbanization. The wetland area has decreased by more than 50% compared to 100 years ago (Belluco et al., 2006; Finlayson and Davidson, 1999; Gallant et al., 2007; Mahdavi et al., 2018). The salt marshes of South Korea have been under constant water pressure from the tidal, and the salt marshes area in Korea has continuously decreased (Cho et al., 2015). In Korea, the conversion of salt marshes to agricultural lands began in the 1930s, and wetlands loss has accelerated further due to additional conversion for industrial development since 1980s (Im et al., 2020).

Recently, mangrove, salt marsh and seagrass ecosystems are globally recognized as major blue carbon sources (Chmura et al., 2003; Duarte et al., 2005; Ewers Lewis et al., 2018; Kristensen et al., 2008). Blue carbon refers to carbon absorbed and stored by vegetation and sediments in marine ecosystems with higher carbon absorption and storage efficiency compared to the terrestrial ecosystems (Lovelock and Reef, 2020; Pendleton et al., 2012). These blue carbon sources absorb carbon dioxide from the atmosphere through photosynthesis and fix carbon into their tissues and sediment (Mcleod et al., 2011). The highest local carbon storage rate value, which is the storage rate of carbon per unit area, was shown in salt marshes (Mcleod et al., 2011). When the comparing net primary production (NPP), the salt marsh had the highest NPP value per unit area among the blue carbon sources (Melillo et al., 1993, Gu et al., 2022). Therefore, the salt marshes have the highest local carbon storage rate and primary productivity among the blue carbon ecosystems and potential for high carbon sequestration (Chmura et al., 2003).

There has been a growing recognition of the importance of salt marsh in providing ecosystem services and sequestering carbon and nitrogen, leading to increased efforts in salt marsh restoration. In salt marshes, the plants can germinate, grow and reproduce in salty soils via physiological mechanisms to remove salt from their tissues (Min, 1998; Waisel, 2012). In Korea, approximately 13 genera of salt marsh plants inhabited (Lee et al., 2019). Among them, *Suaeda japonica*, *Phacelurus latifolius*, *Carex scabrifolia*, *Suaeda glauca* and *Phragmites australis* are commonly found in Korea, with *Su. japonica* being particularly dominant along the west coast (Bang et al., 2018; Lee and Ihm, 2004). *Su japonica* is distributed at the inner side of salt marshes. *Su. japonica* primarily grows in coastal habitats with higher water content and salinity compared to other species (Lee et al., 2009). In addition, the *Suaeda* genus utilizes both C3 and C4 processes that can fix carbon by photosynthesis (Shomer-Ilan et al., 1975). In particular, carbon dioxide fixation by C4 shows high efficiency even in environments with heat, dryness, and salt stress (Haque et al., 2021).

Seedling of salt marsh plant is necessary for salt marsh restoration projects, but research on the growth characteristics of the plants currently found in Korea is very lacking. This study aimed to analyze the potential carbon and nitrogen sequestration capacity of *Su. japonica* by evaluating changes in vegetation and analyzing photosynthetic efficiency, as well as tissue carbon and nitrogen contents of this plant in different seasons and sites. In this study, biomass varied depending on the season and site. The plant density of *Su. japonica* affected survival, growth and photosynthetic efficiency, with the carbon content being higher in stems and roots, while the nitrogen content was higher in leaves.

## 2 Materials and methods

### 2.1 Site description

**Figure 1:**
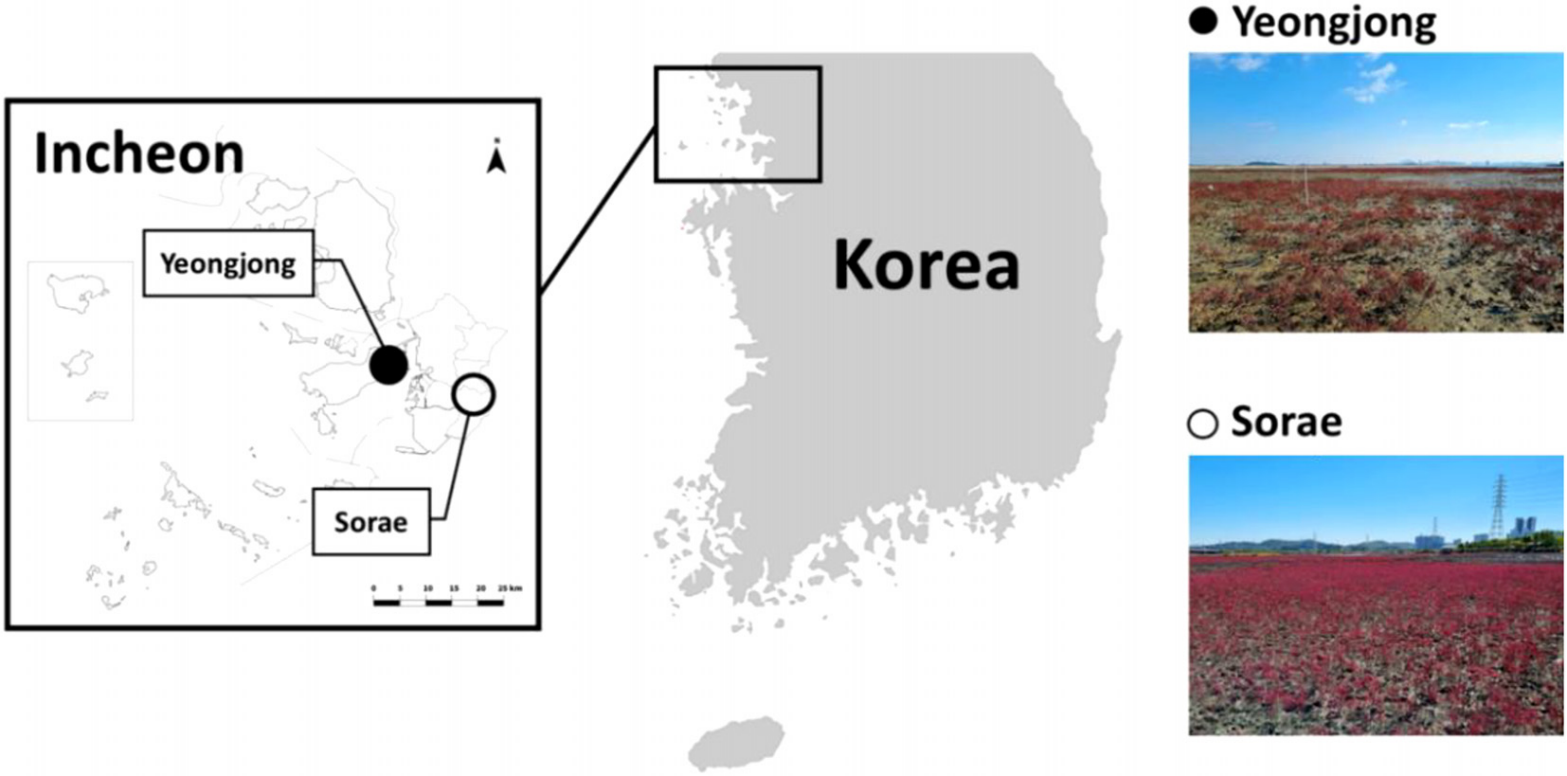
Research sites at Yeongjong Island and Sorae in Incheon, Korea.

Two salt marsh ecosystems in Incheon, Yeongjong Island (N 37°31’11.66520”, E 126°32’31.87320”) and Sorae (N 37°24’31.51079”, E 126°44’46.92480”) were selected based on habitat characteristics and accessibility (**Figure 1**). YJ forms a rias coast, characterized by significant tidal differences of up to 9 m (Lee et al., 2016). At this site, a large-scale tidal flat has been developed, and the sediment layer reaches an average depth of 24 m. The YJ site is affected mostly by seawater with a salinity range of 22.3 to 30.1 (KOEM, 2022). SR is an ecological park created in 1999 by restoring mud flats and abandoned salt farms. The total area of this park is approximately 3,500,000 m^2^. SR is located 7 km away from Seunggi Sewage Treatment Plant and connected to the tidal flow of Jangsu and Seunggee stream and is affected mostly by brackish water (Yoo et al., 2013).

### 2.2 Environmental information by seasons and sites

The sediment analysis was conducted to compare ecological differences by seasons and sites. The annual sedimentation rate was measured using sedimentation plates at each site, and the depth of the sediment accumulated above the plates was measured. In addition, surface sediments were collected and water content and CN content were analyzed. The collected sediment samples were dried to constant weight, and the water content in sediment was calculated from the difference before and after drying. Total organic carbon analysis was performed by treated the samples with 1M HCl to remove inorganic carbon and analyzed using an elemental analyzer. Additional meteorological information (temperature, wind, humidity and isolation) was obtained from the Korea Meteorological Administration (https://minwon.kma.go.kr/main/main.do) for the periods corresponding to the sampling dates: spring (May 1 to 31), summer (July 15 to August 15), and autumn (October 1 to 31) in 2022.

### 2.3 The characteristics of the salt marsh plant stands

The salt marsh vegetation survey was conducted at both sites in different seasons. The survey and sampling in Yeongjong Island and Sorae. were conducted in spring (May 9 and 16, respectively), summer (July 26 and Aug. 3, respectively), and autumn (Oct. 11 and 17, respectively) in 2022. In winter, sampling was conducted on December 22, 2022. However, all plants died and the above-ground parts were damaged, so the winter collection was not included in the results. A quadrat (30 x 30 cm) measurement was performed at 5 randomly selected locations at each site. The photos of quadrats were taken at each site to estimate the percent cover using Image J software (version 1.43, Synoptics, Cambridge, UK).

All salt marsh plants in the quadrat were collected and transferred to the laboratory in a cooler. The collected salt marsh plants were washed with tap water to remove all substance on the surface, and water on the plants was removed using paper towels. The number of salt marsh plants per quadrat was also counted by species to estimate their density. The height of the plant (above- and below-ground) was measured. Then, plants were separated into three parts: root, stem and leaf, and the biomass and height of each part were measured. The fresh weight of each part was measured and the dry weight was measured after drying to a constant weight in a drying oven.


$$Plant\;density\;(plant\;stands/m^{2})=\frac{The\;number\;of\;a\;species}{Total\;area\;of\;quadrat}$$

$$Percent\;cover\;(\%)=\frac{The\;area\;occupied\;by\;a\;species}{Total\;area\;of\;quadrat}*100$$

### 2.4 Species identification

The plants collected from both sites in May, 2022, were washed with distilled water and stored in -20°C for DNA extraction. The DNA extraction was performed using plant DNA/RNA extraction kit (Norgen Biotek Corp., Ontario, Canada). A small piece of leaf, approximately 0.05 g, was ground into powder with liquid nitrogen using pestle and mortar. The extraction and purification of DNA was carried out following the protocol of extraction kit. The integrity of extracted DNA was tested using 1% agarose gel electrophoresis and nanodrop was used for quantitative measurement. DNA sequencing analysis was performed after PCR amplification using the matK gene primer known to show the highest discrimination rate for the identification of Chenopodiaceae species (Yao et al., 2017). The nucleotide sequences obtained from the analysis were identified using BLAST against the GenBank nucleotide Database. Nine individuals were collected from YJ and SR, despite morphological differences such as color and stem shape, all samples were found to be *Suaeda japonica*.

### 2.5 Measurement of photosynthetic efficiency

Photosynthetic efficiency was performed on three leaves at each site using a Diving-PAM (Walz, Effeltrich, Germany). All samples were pre-treated in dark for 20 minutes using a dark adaptation clip and then irradiated with light at 0, 172, 281, 423, 580, 860, 1158, 1735 and 2505 μmol photons m^-2^ s^-1^. The light might be sequentially irradiated for 10 seconds to be expressed as a light reaction curve, and the saturated light was irradiated to measure the quantum yield for each light intensity. Photosynthetic ability of potential chlorophyll was measured through the relative electron transfer rate (rETR). rETR and ETRmax in Photosystem II was calculated using this quantum yield value following the equations below (Beer et al., 1998; Genty et al., 1989; Ralph et al., 1998).


rETR=([F'm−F]/F'm)*PAR*0.5*0.84

ETRmax=ETRs[α/(α+β)][β/(α+β)] β/α

Ik=rETRmax/α

### 2.6 Carbon and nitrogen content in salt marsh plants

All salt marsh plant samples were dried at 60°C in a drying oven to constant weight. The samples were then ground into powder using a Mixer Mill MM 400 (Retsch, Düsseldorf, Germany). Approximately, two to three mg of powder samples were used to analyzed TC (total carbon) and TN (total nitrogen) using an elemental analyzer Flash Smart (Thermo Fisher, Massachusetts, USA).

### 2.7 Carbon and nitrogen sequestration

The amount of carbon and nitrogen sequestration was calculated using dry weight and tissue carbon and nitrogen contents of salt marsh plants. The carbon and nitrogen reduced by salt marsh plants was quantified using the following formula.


$$\begin{array}{c} Carbon\;and\;nitrogen\;sequestration\;of\;salt\;marsh\\ \;plant\;per\;unit\;area\;(\frac{g}{m^{2}})=plant\;dry\;weight(g)\\ /quadrat\;area(m^{2})*carbon/nitrogen\;content(\%) \end{array}$$

### 2.8 Statistical analysis

The statistical analysis for all measurements were performed using SPSS Statistics 25.3.3 (IBM, Armonk, NY, USA). One-way ANOVA and Tukey’s tests were used to confirm *post-hoc* investigation as significant differences (*p* < 0.05). In addition, the plant biomass, carbon and nitrogen content were analyzed using Three-way ANOVA to confirm the complex interactions among plant parts, research sites and seasons (**Supplementary Table S1**).

## 3 Results

### 3.1 Environmental information by seasons and sites

Considering the collection time (10:00-13:00), the weather data at noon was used. The average temperature in spring was 20.0°C, in summer 27.3°C, and in autumn 16.8°C, respectively (**Table 1**). The wind speed was similar by season at 2.7-3.8 m/s. Humidity was higher in summer (79.8%) than spring (50.7%) and autumn (57.1%). Maximum insolation was similar in spring (3.5 MJ m^-2^) and summer (3.3 MJ m^-2^), but average insolation was highest in spring (3.0 MJ m^-2^). The water content of the sediment did not differ significantly by season or location (23.8-27.4%). In spring and summer, total organic carbon (TOC) content of surface sediments was higher in Yeongjong than in Sorae. However, there was no difference between YJ and SR in autumn. The TOC content was also higher in autumn (0.38 and 0.35) compared to spring (0.25 and 0.13) and summer (0.28 and 0.14). The nitrogen content in sediments was not measured below the detection limit of the CN analyzer, 0.01%. The annual sedimentation rate was 1.37 cm yr^-1^ at YJ and 2.53 cm yr^-1^ at SR.

**Table 1:** Environmental parameters in Yeongjong Island (YJ) and Sorae (SR).

Season	Site	Temp (°C)	Wind (m/s)	Humidity (%)	Insolation (MJ m^-2^)	Salinity (psu)	DIN (μg/L)	DIP (μg/L)	Water content in sediment (%)	TOC in sediment (%)	N in sediment (%)	Sedimentation rate (cm yr^-1^)
**Spring**	YJ	20.0 (14.8-23.9)	3.8 (2.2-5.8)	50.7 (17-74)	3.0 (0.8-3.5)	26.60	760.7	19.5	27.4	0.25	ND	1.37
	SR					29.57	419.2	11.64	26.8	0.13	ND	2.53
**Summer**	YJ	27.3 (23.5- 33.9)	3.3 (0.8-8.7)	79.8 (51-98)	2.0 (0.01-3.3)	31.03	294.0	29.3	26.0	0.28	ND	1.37
	SR					30.97	290.5	38.4	23.8	0.14	ND	2.53
**Autumn**	YJ	16.8 (10.3-22.3)	2.7 (1.2-5.5)	57.1 (29-96)	2.1 (0.05-2.6)	27.31	426.4	27.8	26.6	0.38	ND	1.37
	SR					27.78	512.1	45.9	26.6	0.35	ND	2.53

In spring, summer and autumn, water content in sediment (%), total organic carbon (TOC) in sediment (%) and sedimentation rate (cm yr^-1^) were measured in the present study. While temperature (°C), wind (m/s), humidity (%) and insolation (MJ/m^2^) were obtained from the Korea Meteorological Administration (https://minwon.kma.go.kr/main/main.do). Salinity (psu), dissolved inorganic nitrogen (DIN, μg/L), dissolved inorganic phosphorus (DIP, μg/L) were from Incheon Metropolitan City Health and Environment Research Institute (https://www.incheon.go.kr/ecopia/). Annual sedimentation rates (cm yr^-1^) were measured using sedimentation plates at each sites and seasons. ND, Not Detected.

### 3.2 The biomass, cover and height of *S. japonica* stands

The height of the above-ground part was significantly higher in Yeongjong Island (YJ) than in Sorae (SR), while the under-ground part was higher in SR (*p* < 0.001). In spring, the above-ground height of *Suaeda japonica* in YJ was measured at 13.1 cm, while the under-ground part measured at 4.2 cm. The above-ground height of SR was 9.4 cm, with the underground part measuring 6.1 cm (**Figure 1**). Similarly, in summer, the above-ground height of the plants in YJ (30.2 cm) was significantly higher than that of SR (25.1 cm) (*p* < 0.001). However, there was no significant difference by sites in the under-ground part (*p* < 0.05). In autumn, an opposite trend was observed compared to spring. Specifically, the above-ground height of SR (36.1 cm) was significantly higher (*p* < 0.001) than at YJ (32.8 cm), while the under-ground height was significantly higher at YJ (*p* < 0.001).

The biomass of *S. japonica* was measured and divided into roots, stems and leaves. Three-way ANOVA analysis revealed statistically significant differences either individually or in combination with respect to plant part, site, and season (**Supplementary Table S1**). In spring, no significant differences were observed between sampling sites and parts of the plant (*p* < 0.05). Similarly, in summer, there was no difference between SR and YJ, but the biomass of stems and leaves was significantly higher than that of the roots (*p* < 0.001). In autumn, however, all parts in YJ (4.6-32.1 g) showed higher biomass compared to SR (2.2-20.6 g) (*p* < 0.001). The biomass of leaves in YJ (29.0 g) was approximately three times higher than that in SR (10.5 g).

In spring, the plant density per unit area of SR (851.1 plant stands/m^2^) was > 3.5 times higher than that of YJ (235.6 plant stands/m^2^) (*p* < 0.001). However, the density of SR was decreased sharply in autumn (*p* < 0.001), showing no difference from YJ (*p* > 0.05). The plant density of YJ did not show differences by season (*p* > 0.05). However, in terms of percent cover, SR did not show significant differences between the seasons, whereas YJ had a significantly higher value in autumn (23.3%) compared to spring (9.8%) (*p* < 0.001).

### 3.3 Photosynthetic efficiency

**Figure 2:**
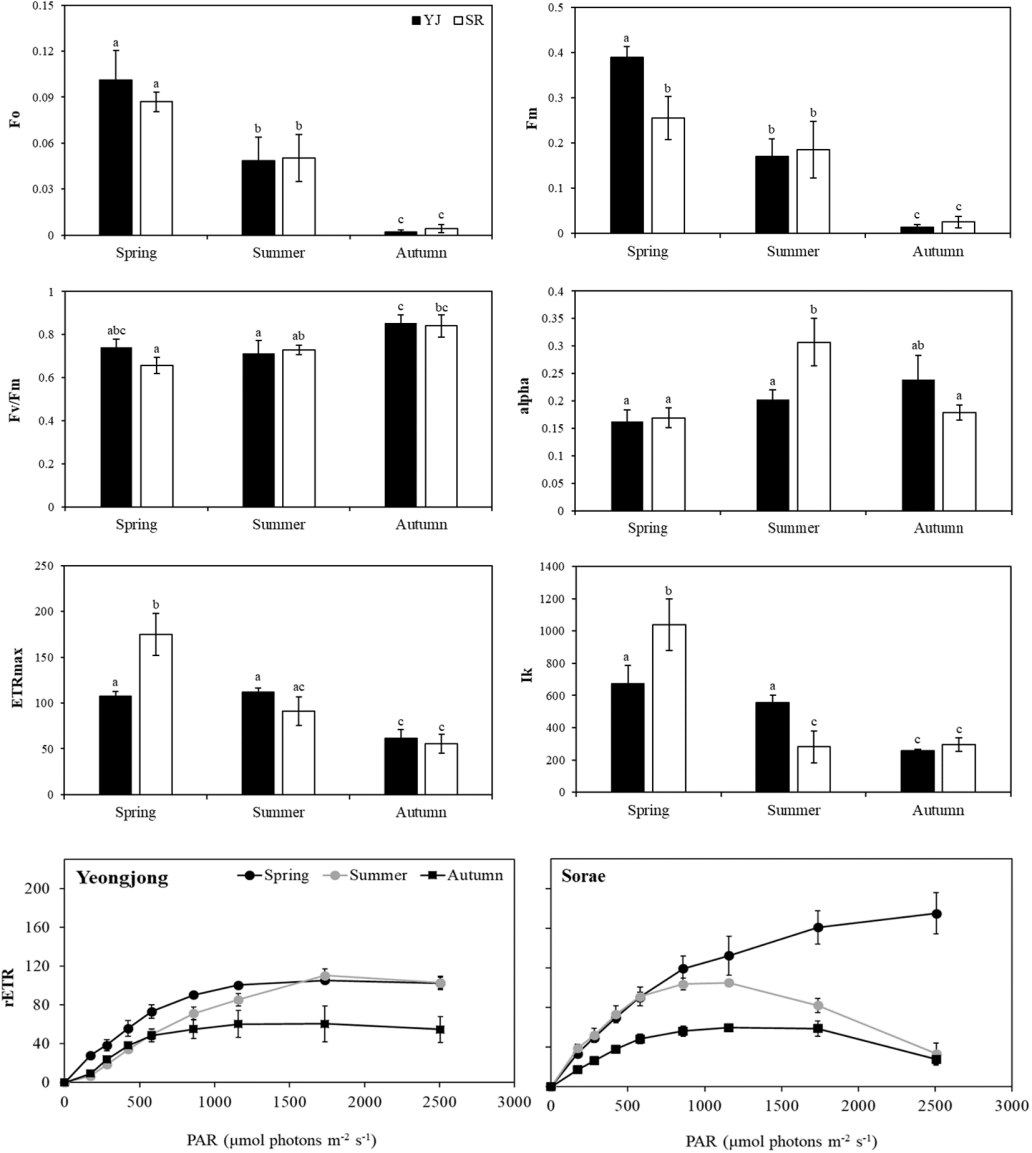
Photosynthetic analysis results of *Suaeda japonica* using a Diving-PAM in different seasons (spring, summer, autumn) and sites (YJ; Yeongjong, SR; Sorae) (Fo; minimum fluorescence, Fm; maximum fluorescence, Fv/Fm; maximum quantum yield, alpha; photosynthetic efficiency, ETRmax; maximum electron transfer rate, Ik; light requirement for saturation of photosynthesis, rETR: relative electron transfer rate). Each coordinate is the overall mean with standard deviation of 3 samples. Different letters on the graph mean significant differences from others (*p* < 0.05).

Photosynthetic analysis using a Diving-PAM was conducted at each site during the peak solar radiation hours (c.a. 10:00 - 13:00). Measurements were taken on clear days and the weather information on those days is summarized in **Table 1**. Since only one species was found at both sites, photosynthetic efficiency was compared in different sites and seasons. When chlorophyll fluorescence was measured after dark adaptation, the minimum fluorescence (Fo) and maximum fluorescence (Fm) did not show significant differences between YJ and SR (*p* < 0.05) (**Figure 2**). However, the values decreased significantly in the order of spring, summer and autumn (*p* < 0.05). Fo and Fm values were close to 0 in autumn. However, the maximum quantum yield (Fv/Fm) did not differ between sites (*p* > 0.05). Only the Fv/Fm value of SR was significantly higher in autumn (0.84) than in spring (0.66) (*p* = 0.02). Photosynthetic efficiency (alpha) was significantly higher in SR in summer (0.31) compared to other seasons and sites (*p* < 0.05). The maximum electron transfer rate (ETRmax) (*p* < 0.001) and light requirement for saturation of photosynthesis (Ik) (*p* < 0.05) were higher at SR than YJ in spring. However, in summer, ETRmax and Ik values significantly decreased at SR (*p* < 0.001). In autumn, ETRmax and Ik of YJ and SR were significantly lower than those in spring (*p* = 0.02). Light saturation occurred at 673 μmol photons m^-2^ s^-1^ in YJ and at 1038 μmol photons m^-2^ s^-1^ in SR in spring. However, the saturated light in SR in autumn (258-295 μmol photons m^-2^ s^-1^) was less than half of than observed in spring.

### 3.4 Tissue carbon and nitrogen contents in salt marsh plants

**Figure 3:**
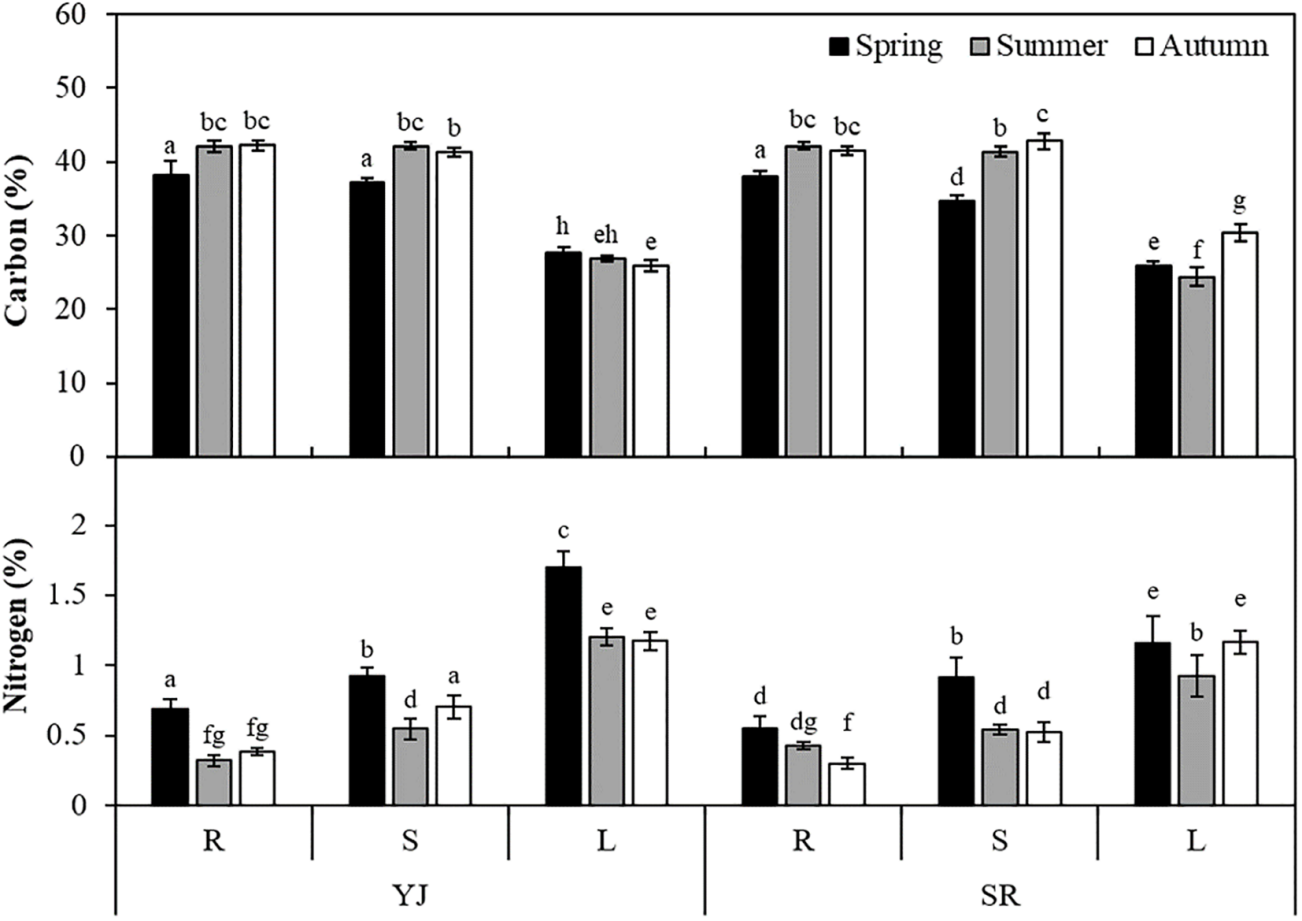
Tissue carbon and nitrogen contents (%) of *Suaeda japonica* measured using an elemental analyzer in different seasons (spring, summer and autumn), sites (YJ; Yeongjong and SR; Sorae) and plant parts (R: roots, S: stems, L: leaves). Different letters on the graph mean significant differences from others (*p* < 0.05).

Tissue carbon content was higher in roots and stems than in leaves in all seasons and sites (*p* < 0.001). Three-way ANOVA analysis indicated that site had a significant effect on carbon content (*p*=0.025), but no significant interaction between site and plant part was observed (*p* > 0.05) (**Supplementary Table S1**). The combination of season, plant part and site affected the carbon content (*p* < 0.001). The carbon contents of roots and stems were approximately 40%, but that of leaves was 27% (**Figure 3**). Both SR and YJ showed higher carbon content in most parts of plant in autumn compared to spring (*p* < 0.001). In particular, the carbon contents in roots and stems were ~ 4% higher in autumn than in spring.

In the results of three-way ANOVA, tissue nitrogen content was affected by study site, plant part, and season, independently or in combination (*p* < 0.001). The highest nitrogen content was observed in leaves, followed by stems and roots, showing an opposite trend to carbon content in all seasons (*p* < 0.05). Nitrogen content was 1.34% in leaves and 0.57% in roots. The nitrogen contents of both YJ and SR were significantly lower in all plant parts in autumn compared to spring (*p* < 0.05).

## 4 Discussion

Living in salt marshes requires the ability to adapt to seasonal fluctuations in salinity as well as other environmental challenges such as precipitation, evaporation, and seawater inflow (Waisel, 2012). First of all, while temperature and humidity varied with the seasons, insolation, wind and sediment water content remained unchanged (**Table 1**). In addition, the dissolved inorganic nitrogen (DIN) dissolved inorganic phosphorus (DIP), and salinity were measured by Incheon Metropolitan City Health and Environment Research Institute (https://www.incheon.go.kr/ecopia/) at the nearest locations, approximately 2 and 9 km from YJ and SR, respectively. The DIN, DIP and salinity of seawater flowing into YJ and SR showed no differences in summer and autumn. However, in spring, the DIN concentration was nearly twice as high in YJ (760.7 μg/L) compared to SR (419.2 μg/L). On the other hand, the present study revealed that the plant density at SR was 851 individuals m^-2^ in spring and significantly decreased to 213 individuals m^-2^ in autumn. In contrast, YJ, which had a lower initial plant density did not show any significant changes between spring and autumn. The observed difference in plant density between YJ and SR is likely attributed to variations in seawater inflow rather than other environment conditions. The YJ site is directly affected by tidal action, with the seawater flowing in and out twice a day. In contrast, the SR site, an artificial wetland ecological park experiences flooding only when the high tide level exceeds 9 m, occurring approximately 2-3 times per month (Park et al., 2023).

**Figure 4:**
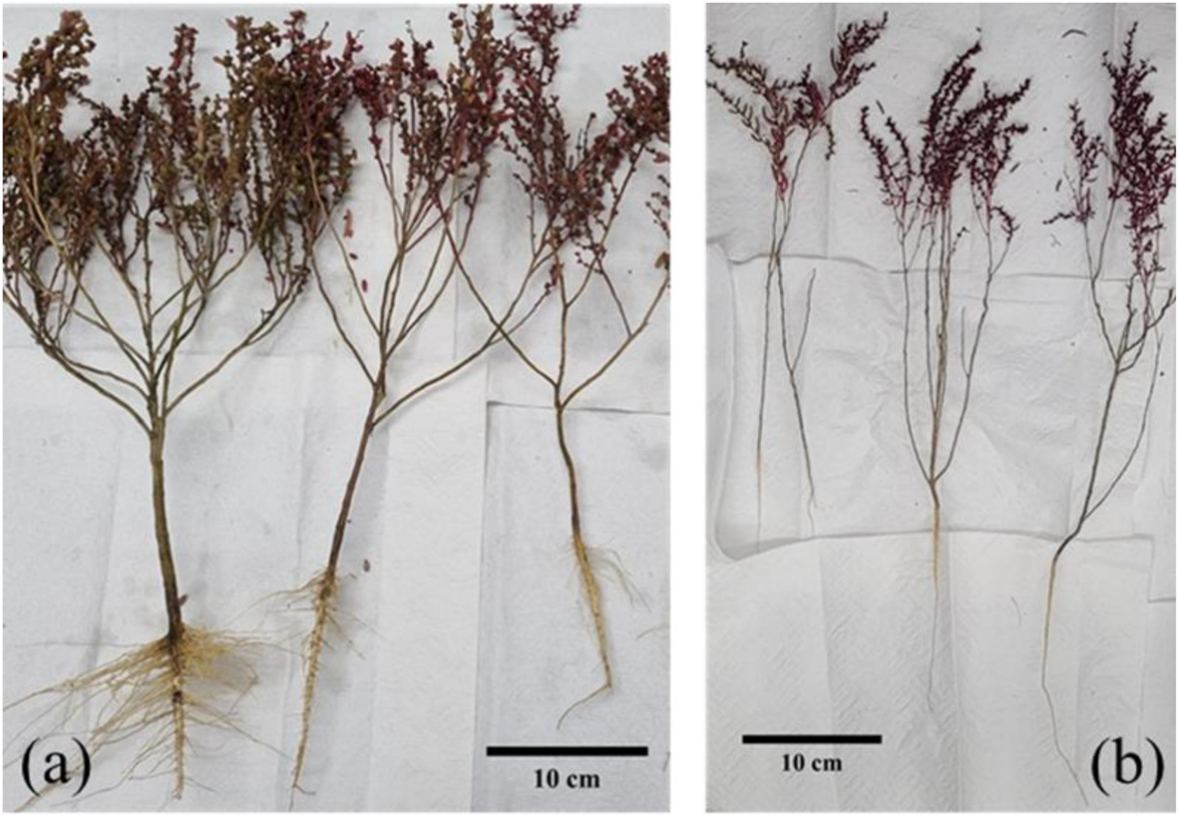
*Suaeda japonica* showing morphological differences in autumn 2022 (**a**: Yeongjong, **b**: Sorae).

Plant density also affects the plant survival and growth (Johnson et al., 2017; Comita et al., 2014; Terborgh, 2012; Lu et al., 2015). In general, as plant density increases, the number of branches decreases (Leach et al., 1999). In this study, *S. japonica* of SR grew thinner and elongated, whereas the plants at YJ grew thicker and wider with more branches (**Figure 4**). A previous study was conducted to determine changes in the biomass of *Suaeda salsa* in relation to plant density (Xu et al., 2023). Initially, when the plant density was 30, 40, 50 and 60 plants m^-2^, the height of *S. salsa* increased with plant density. However, as the plants grew, their height, stem diameter, canopy width and biomass decreased with increasing plant density (Xu et al., 2023).

Photosynthesis was also affected by the plant density. In summer, the values of ETRmax and Ik of *S. japonica* significantly decreased at SR compared to spring. SR showed lower light saturation than YJ in both summer and autumn. The lower photosynthetic rate is due to light interruption by higher plant density, which reduces the electron transfer rate and light saturation. For example, *Atriplex prostrata*, an annual plant in the Amaranthaceae family, showed increased intraspecific competition at a higher plant density (Wang et al., 2005). The net photosynthetic rate of this plant decreased from 7.6 ± 0.9 μmol CO_2_ m^-2^ s^-1^ at a lower plant density to 3.5 ± 0.4 μmol CO_2_ m^-2^s^-1^ at a higher density (Wang et al., 2005). Short-term light inhibition reduces electron transfer rate or light saturation, while long-term light restrictions can inhibit plant growth, leaf area expansion, leaf gas exchange and biomass increase (Ballaré et al., 1994; Ren et al., 2017; Wells, 1991).

In Yeongjong (YJ) and Sorae (SR) salt marshes, seasonal changes were observed from May to October (**Table 1**). In addition, *Suaeda japonica* showed some morphological changes across seasons. In spring, when new plants begin to grow, the stems and leaves are green and red. As summer and autumn progresses, the plant develops many branches and turns predominantly red. This color change is due to the accumulation of betacyanin, a red pigment, in the plants during summer to autumn (Hayakawa and Agarie, 2010; Akbar Hussain et al., 2018; Azeredo, 2009). Hayakawa and Agarie (2010) reported that in *S. japonica*, chlorophyll content sharply decreased in autumn compared to summer, while betacyanin contents increased significantly in autumn. As the chlorophyll content of the leaves decreases, photosynthetic efficiency can also be significantly reduced.

**Figure 5:**
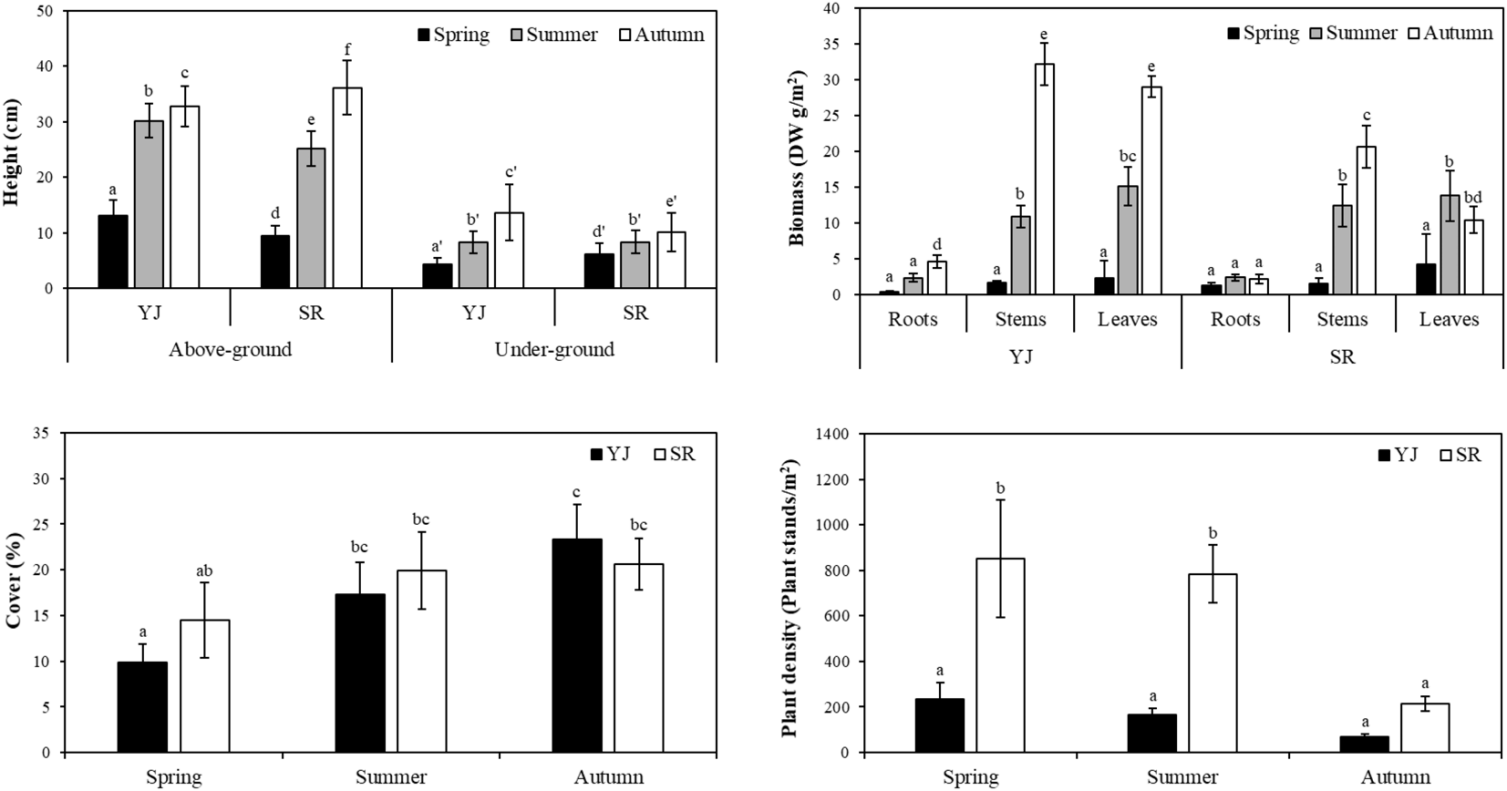
The biomass, cover and height of *S. japonica* stands of *Suaeda japonica* measured in different sites (YJ: Yeongjong and SR: Sorae) and seasons (spring, summer and autumn). The plant height (cm) of above- and under-ground, biomass of each part (roots, stems and leaves) per unit area, and plant density (plant stands/m^2^) and coverage (%) were measured. Different letters on the graph mean significant differences from others (*p* < 0.05).

Salt marshes called “blue carbon”, absorbed carbon through primary production and plays an important role in sequestering carbon dioxide in sediment (Duarte et al., 2013). In this study, the dry weight of the underground parts was 7-19% of whole plant (**Figure 5**). Photosynthetic rates were higher in spring and summer than in autumn, but the carbon content in stems and roots (41.3-42.8%) were higher in autumn than in spring (34.7-38.1%). These results suggest that carbon absorbed through photosynthesis in leaves is transferred to and stored in stems and roots. In **Table 1**, when the TOC content of surface sediments was analyzed by seasons, a higher content (0.35-38%) was observed in the autumn as biomass of plants was high. Additionally, sedimentation rates of 1.53 and 2.53 cm were observed annually in YJ and SR, respectively. As a result, the carbon stored in the underground parts and dead tissue of the plants can be sequestered in sediments.

Tissue carbon content was higher in the roots (34.7-42.8%) compared to other parts, while nitrogen content was significantly higher in leaves (0.92-1.70%) than in the roots and stems (0.32-0.92%). Both YJ and SR showed higher nitrogen content in all part in spring than other seasons (*p* < 0.05). In spring, the nitrogen content of leaves in YJ was higher than at SR (*p* < 0.001), but there was no significant difference in autumn (*p* > 0.05). Cartaxana and Catarino (1997) compared nitrogen content in different parts of *Spartina maritima*, *Halimione portulacoides* and *Arthrocnemum perenne* and found higher nitrogen content in the leaves probably because leaves are the major plant part for photosynthesis. These results suggest that chlorophylls in leaves are a major storage for nitrogen. When *S. japonica* dies in late autumn, the stored nitrogen in its tissues can be buried underground and accumulated in sediments.

Carbon sequestration by *S. japonica* did not differ between sites in spring and summer. In autumn, however, the amount of carbon sequestration by *S. japonica* was much higher at YJ (114 g C m^-2^) than SR (65 g C m^-2^) (**Table 2**). Nitrogen sequestration also did not differ in spring and summer, but the nitrogen sequestration of YJ (3.0 g N m^-2^) was higher compared to SR (1.2 g N m^-2^). The area of the salt marsh ecosystem at YJ and SR is approximately 3,905,000 m^2^ and 3,500,000m^2^, respectively. Considering these areas, along with the tissue carbon and nitrogen contents of the plants, the total carbon and nitrogen sequestration is estimated to be approximately 440 (1,630 CO_2_) tons and 11 tons, respectively at YJ and 230 (830 CO_2_) tons and 4 tons, respectively at SR. These values are very significant, approximately 136.0% of total organic carbon and 1.6% of total nitrogen emitted by the closest wastewater treatment plant (WTP) from SR, Seunggee WTP, Incheon in 2022 (Environmental Corporation of Incheon, 2024).

**Table 2:** The amount of carbon and nitrogen sequestration by *Suaeda japonica* in different seasons at Yeongjong Island and Sorae.

			Sequestration (g m^-2^)	
		Dry weight (g m^-2^)	Carbon (CO_2_)	Nitrogen
**Spring**	**YJ**	22.43 ± 3.98 ^a^	7.24 ± 1.15 ^a^ (26.57)	0.29 ± 0.04 ^a^
	**SR**	35.51 ± 6.47 ^a^	10.69 ± 1.66 ^a^ (39.23)	0.35 ± 0.09 ^a^
**Summer**	**YJ**	142.24 ± 22.70 ^b^	48.50 ± 7.92 ^b^ (117.98)	1.25 ± 0.20 ^b^
	**SR**	143.57 ± 33.43 ^b^	47.63 ± 10.43 ^b^ (174.81)	1.02 ± 0.21 ^b^
**Autumn**	**YJ**	329.0 ± 22.89 ^c^	113.70 ± 9.91 ^c^ (417.27)	2.95 ± 0.30 ^c^
	**SR**	166.47 ± 25.97 ^b^	64.77 ± 10.05 ^d^ (237.70)	1.17 ± 0.16 ^b^

Different letters on the graph mean significant differences from others (*p* < 0.05).

## 5 Conclusion

Yeongjong (YJ) experiences tidal inflow and outflow twice a day, whereas Sorae (SR), an artificial ecological park, is affected by tides only 2-3 times a month. The differences in seawater inflow may have influenced the plant density and biomass of *Suaeda japonica*. Consequently, in spring, the plant density at SR was approximately four times higher than at YJ. However, the higher plant density led to morphological variations and inhibited the survival, growth and photosynthetic efficiency of *S. japonica*. At SR, both the plant density and photosynthetic efficiency significantly decreased in summer compared to spring. *S. japonica* at YJ (lower density) grew wider with many branches, while at SR (higher density), the plants grew longer with fewer branches. These morphological differences were reflected in their carbon sequestration capacity. YJ exhibited a higher carbon sequestration per unit area, approximately 113.70 g/m², which was twice that of SR. Tissue nitrogen content was higher in leaves than in roots or stems, while tissue carbon content was higher in roots and stems than in leaves. The high carbon content in roots and stems suggest that carbon absorbed via photosynthesis in leaves can be transferred and stored underground. This study emphasizes the critical role of salt marshes as blue carbon resources and advocates for continued and enhanced restoration efforts. By quantifying the carbon sequestration capabilities of *S. japonica*, we provide valuable insights that support the need for targeted conservation and restoration projects to mitigate climate change impacts.

## Data Availability

The raw data supporting the conclusions of this article will be made available by the authors, without undue reservation.
