# Supplementary material for: Seasonal variations in biomass, height, photosynthetic efficiency, and carbon and nitrogen contents of Suaeda japonica in Incheon salt marshes (Korea)
Source: Front Plant Sci. 2025 Mar 11;16:1513624. doi: 10.3389/fpls.2025.1513624 (PMC11933049; doi:10.3389/fpls.2025.1513624)
Supplement: Supplementary file 1 [file Table1.pdf]

Supplementary Tables

Supplementary table 1.

Results of three-way ANOVA examining the effects of plant parts (roots, stems and leaves), research sites (Yeongjong and Sorae) and seasons (spring, summer and autumn) on plant weight, carbon content and nitrogen content. This statistical analysis was performed using SPSS Statistics 25.3.3 (IBM, Armonk, NY, USA).

Variable	Factor	F	p-value
**Weight**	site	31.761	**<0.001**
	season	207.959	**<0.001**
	part	147.791	**<0.001**
	site * season	41.850	**<0.001**
	site * part	7.429	**<0.001**
	season * part	44.230	**<0.001**
	site * season * part	7.404	**<0.001**
**Carbon content**	site	5.092	**0.025**
	season	335.874	**<0.001**
	part	5222.953	**<0.001**
	site * season	64.856	**<0.001**
	site * part	2.235	0.108
	season * part	96.242	**<0.001**
	site * season * part	34.596	**<0.001**
**Nitrogen content**	site	110.464	**<0.001**
	season	287.739	**<0.001**
	part	1434.584	**<0.001**
	site * season	18.636	**<0.001**
	site * part	39.408	**<0.001**
	season * part	5.443	**<0.001**
	site * season * part	27.783	**<0.001**
